# In vitro toxicity of particulate matter (PM) collected at different sites in the Netherlands is associated with PM composition, size fraction and oxidative potential - the RAPTES project

**DOI:** 10.1186/1743-8977-8-26

**Published:** 2011-09-02

**Authors:** Maaike Steenhof, Ilse Gosens, Maciej Strak, Krystal J Godri, Gerard Hoek, Flemming R Cassee, Ian S Mudway, Frank J Kelly, Roy M Harrison, Erik Lebret, Bert Brunekreef, Nicole AH Janssen, Raymond HH Pieters

**Affiliations:** 1 Division of Toxicology and Division of Environmental Epidemiology, Institute for Risk Assessment Sciences (IRAS), Utrecht University, P.O. Box 80177, 3508 TD Utrecht, The Netherlands; 2 Centre for Environmental Health, National Institute for Public Health and the Environment (RIVM), P.O. Box 1, 3720 BA Bilthoven, The Netherlands; 3 MRC-HPA Centre for Environment and Health, School of Biomedical Sciences, King's College London, 150 Stamford Street, London SE1 9NH, UK; 4 Division of Environmental Health & Risk Management, School of Geography, Earth & Environmental Sciences, University of Birmingham, Edgbaston, Birmingham B15 2TT, UK; 5 Julius Center for Health Sciences and Primary Care, University Medical Center Utrecht, P.O. Box 85500, 3508 GA Utrecht, The Netherlands

## Abstract

**Background:**

Ambient particulate matter (PM) exposure is associated with respiratory and cardiovascular morbidity and mortality. To what extent such effects are different for PM obtained from different sources or locations is still unclear. This study investigated the *in vitro *toxicity of ambient PM collected at different sites in the Netherlands in relation to PM composition and oxidative potential.

**Method:**

PM was sampled at eight sites: three traffic sites, an underground train station, as well as a harbor, farm, steelworks, and urban background location. Coarse (2.5-10 μm), fine (< 2.5 μm) and quasi ultrafine PM (qUF; < 0.18 μm) were sampled at each site. Murine macrophages (RAW 264.7 cells) were exposed to increasing concentrations of PM from these sites (6.25-12.5-25-50-100 μg/ml; corresponding to 3.68-58.8 μg/cm^2^). Following overnight incubation, MTT-reduction activity (a measure of metabolic activity) and the release of pro-inflammatory markers (Tumor Necrosis Factor-alpha, TNF-α; Interleukin-6, IL-6; Macrophage Inflammatory Protein-2, MIP-2) were measured. The oxidative potential and the endotoxin content of each PM sample were determined in a DTT- and LAL-assay respectively. Multiple linear regression was used to assess the relationship between the cellular responses and PM characteristics: concentration, site, size fraction, oxidative potential and endotoxin content.

**Results:**

Most PM samples induced a concentration-dependent decrease in MTT-reduction activity and an increase in pro-inflammatory markers with the exception of the urban background and stop & go traffic samples. Fine and qUF samples of traffic locations, characterized by a high concentration of elemental and organic carbon, induced the highest pro-inflammatory activity. The pro-inflammatory response to coarse samples was associated with the endotoxin level, which was found to increase dramatically during a three-day sample concentration procedure in the laboratory. The underground samples, characterized by a high content of transition metals, showed the largest decrease in MTT-reduction activity. PM size fraction was not related to MTT-reduction activity, whereas there was a statistically significant difference in pro-inflammatory activity between Fine and qUF PM. Furthermore, there was a statistically significant negative association between PM oxidative potential and MTT-reduction activity.

**Conclusion:**

The response of RAW264.7 cells to ambient PM was markedly different using samples collected at various sites in the Netherlands that differed in their local PM emission sources. Our results are in support of other investigations showing that the chemical composition as well as oxidative potential are determinants of PM induced toxicity *in vitro*.

## Background

Ambient particulate matter (PM) is associated with morbidity and mortality due to respiratory and cardiovascular disease [1-4]. Several mechanisms have been proposed to explain PM related health effects. In the past few years, the ability of PM to induce inflammatory effects [5-7] as well as oxidative stress derived effects [8-10] has been demonstrated repeatedly. To what extent such effects are different for PM obtained from different sources or locations is still unclear.

Inflammation is characterized by local recruitment of pro-inflammatory cells such as neutrophils and macrophages, which leads to increased production of cytokines and chemokines such as tumor necrosis factor alpha (TNF-α), interleukin (IL)-1, -6, and -8. Furthermore, pro-inflammatory cells, such as neutrophils and macrophages, are well known to generate reactive oxygen species (ROS) which could participate in the generation of cellular stress by PM. Oxidative stress could also be directly generated by PM, since several compounds absorbed on the particles surface are redox-active or may become redox-active after biotransformation *in vivo *[10,11].

Variations in the physical and chemical composition of ambient PM may influence the pro-inflammatory and oxidative potential [12,13]. The composition of PM is determined by emissions from different sources such as traffic, industry, agricultural activities, and long distance transported air pollution. These emissions are affected by seasonal variations in temperature and other meteorological conditions implicated in atmospheric transformation processes. Although there is a large amount of data available on the toxicity of ambient PM there is limited information on the influence of PM chemical components in relation to sources and origin of PM [14].

So far, several components have been associated with adverse effects of PM. For example, it has been demonstrated that both water soluble transition metals and endotoxin (i.e. a component of the outer membrane of Gram-negative bacteria) can induce a pro-inflammatory effect [15-19]. Transition metals are also considered to contribute to PM induced cytotoxicity and oxidative stress [20]. In addition to metals, organic components of PM, such as polycyclic aromatic hydrocarbons (PAHs) and their oxygenated derivatives (e.g. quinones) may also play a role in oxidative stress [8,21,22]. More insight in the relative contribution of PM components to adverse health effects would enable a more straightforward approach to air quality management focused on specific components and sources, rather than on PM mass concentration which is currently used for air quality legislation.

The objective of this study was to investigate the *in vitro *toxicity of particulate matter collected at different sites, influenced by different emission sources, in the Netherlands in relation to PM composition and oxidative potential. This study is part of the RAPTES project: Risk of Airborne Particles - a hybrid Toxicological and Epidemiological study. The aim of RAPTES is to characterize the physical, chemical and oxidant properties of inhaled PM and to establish which of these characteristics determine the adverse respiratory and systemic effects seen after PM exposures. Eight sampling sites across the Netherlands were chosen to represent different PM emission sources relevant for human exposure. Size-segregated PM was collected at each location for *in vitro *and *in vivo *experiments as well as a detailed chemical characterization. This paper describes the results of *in vitro *exposures of immortalized mouse macrophages to PM samples collected during RAPTES.

## Methods

### PM collection

#### Sampling period

Ambient coarse (2.5-10 μm), fine (< 2.5 μm), and quasi ultrafine (qUF; < 0.18 μm) PM was collected at eight sites in the Netherlands during the period June 2007 - February 2008. PM sampling was carried out for 3 - 4 separate days and for 6 hours per day at each site (between 09:00-16:00 h). Per site, the sampling day with highest PM concentration (μg/ml; see sampling procedure) was chosen for further analysis to ensure sufficient availability of material for the various tests.

#### Sampling sites

The sampling sites were selected based on expected high contrast and low correlation between major PM components and contributing PM sources. A detailed description of the sampling sites and the air quality during the sampling campaign is described elsewhere [23]. Briefly, sites include an urban background site (a semi-enclosed courtyard located in the city centre of Rotterdam), a farm site (an organic farm located in a rural area), a continuous traffic site (next to motorway A15), a stop-and-go traffic site (next to a traffic intersection in Rotterdam), an underground train station (platform of a train station located in a train tunnel), a truck traffic site (next to a secondary road, primarily used by diesel trucks), a harbor site (next to a shipping canal to the port of Rotterdam), and a steelworks site (about 2.5 km downwind from a steel mill).

#### Sampling procedure

PM for this *in vitro *study was collected using the Versatile Aerosol Concentration Enrichment System (VACES), using three parallel sampling lines (concentrators) to simultaneously collect coarse, fine, and quasi ultrafine particles at a flow rate of 110 L min^-1^. The VACES is described in greater detail by Kim *et al*. [24,25]. Briefly, coarse particles were concentrated using a single-nozzle virtual impactor. Fine and qUF particles were concentrated by drawing air samples through two parallel lines, using 2.5 μm and 0.18 μm cut-point pre-impactors respectively, to remove larger-sized particles. The VACES thus collects a quasi-ultrafine PM size fraction (PM with an aerodynamic diameter, Dp < 0.18 μm), a fine fraction (Dp < 2.5 μm, so including ultrafine particles) and a coarse fraction (2.5 < Dp < 10 μm). Both of the smaller size fractions are drawn through a saturation-condensation system that grows particles to 2-3 μm droplets, which are subsequently concentrated by virtual impaction. Highly concentrated liquid suspensions of all three particle modes were obtained by connecting the concentrated output flow from each of the VACES concentrators to a liquid impinger (BioSampler, SKC West Inc., Fullerton, CA). Since coarse particles are capable of penetrating directly into water, and therefore do not need to be drawn trough a saturation-condensation system, the impinger for the coarse mode was filled with fresh distilled water before each use. The VACES and the BioSampler were cleaned prior to use with pure methanol.

### PM preparation

The PM concentration of the collected samples ranged from 12 μg/ml (urban background, fine) to 677 μg/ml (Underground, coarse). To enable *in vitro *testing on equal PM mass concentration, samples were concentrated by evaporation of water in PM samples or diluted to 200 μg/ml. For concentration purposes 1 ml aliquots of PM suspensions were placed in a heating block (25°C) under a constant nitrogen flow (99.5% nitrogen) for 0 to 5 days depending on the amount of water that had to be evaporated. PM samples with a concentration of more than 200 μg/ml at start (all underground samples) were diluted in pyrogen-free water. The start concentration of 200 μg/ml was chosen to obtain a final concentration range of 6.25 to 100 μg/ml based on a review of Mitschik *et al*. [26].

### PM characterization

#### Air pollutants and meteorological conditions

Next to VACES, an array of PM sampling equipment was used during each sampling day as described in detail by Strak *et al*. [23]. Gaseous air pollutants, nitrogen (di)oxide and ozone, were measured using real-time monitors from Thermo Environmental Instruments Inc., Franklin, USA (chemiluminescence NO-NO_2_- NO_x _analyzer model 42 and U.V. Photometric O3 Analyzer Model 49, respectively). The particle number concentration (PNC) was measured with a real-time condensation particle counter (CPC model 3022A; TSI, St Paul, MN). In addition, a weather station was used to measure meteorological conditions during the sampling days (Davis VantagePro2; Davis Instruments, Hayward, CA).

#### Chemical composition

A high-volume sampler (HVS; model TE-6070V equipped with TE-231 High Volume Cascade Impactor, Tisch Environmental, Cleves, USA) was used to sample both coarse and fine PM on quartz microfiber filters. PM was extracted from the quartz filters and subsequently analyzed on chemical composition. Detailed extraction and analysis procedures are described elsewhere [23]. PM mass concentration was measured by using a Micro-Orifice Uniform Deposit Impactor (MOUDI; MSP Corporation, Minneapolis, USA) with three, non-rotating stages (10-2.5, 2.5-1.8 and < 1.8 μm). Chemical composition data (HVS; μg/m^3^) was divided by PM mass concentration data (MOUDI; μg/m^3^) to obtain chemical composition per μg PM.

#### Oxidative potential and endotoxin content

Oxidative potential as well as endotoxin content was assessed on VACES samples that were used for the *in vitro *toxicity testing. The oxidative potential of the concentrated PM samples was determined using a modified DTT assay [21] as described in Additional file 1. Oxidative potential (DTT consumption) is expressed as the rate of DTT consumption which is normalized to the quantity of PM used. Endotoxin levels in the concentrated PM samples were assayed using a quantitative kinetic chromogenic Limulus Amoebocyte Lysate (LAL) test kit according to manufactures instructions (Lonza Sales Ltd, Basel, Switzerland). The LAL assay provides an indication for the endotoxin content, Spaan *et al*. [27] reported an average coefficient of variation of 20% for duplicate samples.

### In vitro exposure

#### Cell culture

The murine macrophage cell line RAW 264.7 was obtained from American Type Culture Collection (ATCC, Rockville, MD, USA). Cells were cultured and maintained according to the ATCC protocol with minor modifications (Additional file 1).

#### Experimental setup

Cells were seeded at 1 × 10^5 ^cells per well (100 μl/well) in 96-well plates (cell culture area 0.34 cm^2^/well; Greiner-Bio-One, Solingen Germany) and allowed to adhere for approximately 6 hours. Two-fold serial dilutions (6.25-100 μg/ml; corresponding to 3.68 - 58.8 μg/cm^2^) of the PM samples were prepared in DMEM/F12 powder medium (GIBCO Invitrogen, Breda, The Netherlands) The latter was prepared according to manufacturer's instructions and replenished with 10% FCS (Greiner Bio-One, Solingen, Germany), 1% penicillin-streptomycin (GIBCO Invitrogen, Breda, The Netherlands). All PM samples were sonicated (Branson 1510 Ultrasonic bath; Branson, Danbury, USA) for approximately 30 seconds before each use to suspend the PM into their medium. Subsequently, the freshly prepared PM solutions were added to the cells in a maximum volume of 100 μl per well. Following an overnight exposure of 16 hours, the supernatant was removed and stored at -20°C until for further analysis (cytokine levels by means of ELISA). The cells were used in the MTT assay as described below.

For each PM sampling site one 96-well plate was used containing the different size fractions of PM dilutions in triplicate. Furthermore, each plate also had a negative (untreated cells) and a positive control (cells treated with Lipopolysaccharide [LPS], LPS induces pro-inflammatory cytokine production in RAW 264.7 macrophages). On each plate, cells were exposed to an increasing concentration (3, 10 and 30 ng/ml) of LPS (O111:B4, Sigma-Aldrich, Zwijndrecht, the Netherlands) in triplicate wells. There was relatively little plate-to-plate variability in LPS induced cellular responses. The CV was smaller than 35% for all parameters measured with exception of IL-6 (16 plates, n = 48 wells; MTT-reduction activity CV ≤ 17%; TNF-α CV ≤ 29%; MIP-2 (CV ≤ 34%; IL-6 CV 50-93%). In total, two independent experiments were performed.

#### MTT assay

The MTT assay was used as a measure of metabolic activity and performed as described by Mosmann [28] with minor modifications (Additional file 1). MTT-reduction activity was expressed as percentage of the unexposed control cells (100%).

#### ELISA's

Cytokine and chemokine levels were determined in the supernatant. The cytokines TNF-α and IL-6 were immunochemically analyzed using commercial available enzyme-linked immunosorbent assay (ELISA) kits (eBioscience, Vienna, Austria). Furthermore, the chemokine MIP-2 was determined using an ELISA kit of KOMA Bio-tech. (KOMA Bio-tech., Seoul, Korea). All ELISA's were performed according to manufacturer's instructions.

### Statistical analysis

We assessed the concentration-response relationships between PM exposure and the different cellular responses (MTT-reduction activity, cytokines and chemokine production) using multiple linear regression. The same regression analyses were performed for assessing the associations between cellular responses and PM size fraction, PM oxidative potential (DTT consumption) and endotoxin content. The general equation of the models tested was: Y = b0 + b1*PMconcentration + b2*X +b3*(X*PMconcentration) + E, where × was either sampling site, PM size fraction, PM oxidative potential or PM endotoxin content. Since the underground train station site was the only non-outdoor sampling location, PM characteristics like chemical composition and particle size distribution were expected to be different from the other (outdoor) sites. To investigate the influence of underground train station PM on the relation between outdoor collected PM and *in vitro *toxicity, we repeated all regression analyses after excluding the underground train station site. All the differences were considered to be statistically significant at the p < 0.05 level. The data were analyzed using SAS version 9.2 (SAS Institute Inc, NC, USA).

## Results

### Air pollution characteristics

Characteristic features of air pollution for each site during the sampling day are shown in Table 1. For all PM size ranges, the lowest mass concentrations were measured at the urban background site, whereas the highest were measured at the underground train station site. The median particle number concentration ranged from 6,700 (farm) to 64,700 particles/cm^3 ^(stop & go traffic). Background levels of PM_10_, obtained from the Dutch National Air Quality Monitoring Network [23], were highest during the sampling day at the stop & go traffic site (90 μg/m^3^). During the other sampling days, the background levels were lower (13 - 27 μg/m^3^).

**Table 1:** Mean values of air pollutants and meteorological conditions at the selected sampling days

Site	Date	PM_10-2.5 μm_	PM_2.5-0.18 μm_	PM_0.18 μm_	PNC	O_3_	NO	NO_2 _	Temp	RH	Wind	BG PM_10 _
		**(μg/m^3^)**	**(μg/m^3^)**	**(μg/m^3^)**	**(#/cm^3^)**	**(ppb)**	**(ppb)**	**(ppb)**	**(°C)**	**(%)**	**(m/s)**	**(μg/m^3^)**

Farm	05-Jul-07	4.3	6.7	3.8	6700	30	2.99	6.6	17	81	5.3	18

Urban background	02-Jul-07	3.9	6.1	2.0	20600	23	7.39	16	18	81	0.26	15

Steelworks	16-Jul-07	6.0	11	4.5	NA	37	14.1	32	21	84	1.2	27

Harbor	13-Dec-07	6.1	19	5.5	19600	3.9	25.2	24	3.8	90	3.2	24

Continuous traffic	12-Jul-07	15	16	4.4	NA	12	36.5	25	18	79	2.5	26

Truck traffic	26-Jul-07	6.0	14	4.1	42800	4.5	106	30	20	81	3.8	17

Stop & go traffic	24-Jan-08	43	28	13	64700	2.4	41.1	38	11	90	4.8	90

Underground	26-Sep-07	58	38	83*	39300	0.9	14.9	20	18	57	0.00	13

PNC, particle number concentration (median); Temp, temperature; RH, relative humidity; BG, background levels of PM_10_; NA, not available; * selected day not available, mass concentration estimated based on average values during other sampling days at this site.

### Chemical composition

Coarse and fine filter extracts from a HVS were used for an in-depth chemical analysis (Table 2 and 3). Data of the farm and urban background site was not available due to equipment failure on the sampling days chosen for the *in vitro *toxicity testing. For coarse PM, the highest metal content was found in PM collected at the underground train station site. The most abundant species were iron (305 ng per μg PM) and copper (27 ng per μg PM). PAH were most abundant in the steelworks sample (34 pg per μg coarse PM) followed by the underground sample (29 pg per μg coarse PM). In the fine size range, contrasts in metal content between sites followed roughly the same pattern as for the coarse samples. Again, the highest metal content was found in PM collected at the underground train station site with iron and copper being the most dominant species (464 and 41 ng per μg fine PM respectively). Elemental carbon (EC) and organic carbon (OC) were highest in the continuous traffic and the truck traffic samples (EC 0.96, OC 0.57 and EC 1.02, OC 0.42 μg per μg PM respectively). The apparent discrepancy in chemical mass closure (e.g. OC/EC ~ 1 μg per μg PM) is caused by using different equipment to collect PM for chemical composition and PM for mass concentration determination.

**Table 2:** Chemical composition of coarse particulate matter (PM) collected at the selected sampling days

Coarse PM		Trace metals						Soluble inorganic compounds				Organic compounds		
**(2.5-10 μm)**		**(ng/μg PM)**						**(μg/μg PM)**				**(μg/μg PM)**		**(pg/μg PM)**

**Site**	**Sampling day**	**Al**	**Cu**	**Fe**	**Ni**	**V**	**Zn**	**NH_4_^+^**	**Cl^-^**	**NO_3_^-^**	**SO_4_^2-^**	**EC**	**OC**	**PAH**

Steelworks	16-Jul-2007	2.2	0.07	11	0.03	0.16	0.30	0.01	0.02	0.05	0.02	0.05	0.16	34

Harbor	13-Dec-2007	0.82	0.30	6.1	0.02	0.03	-	0.02	0.00	0.05	0.02	0.01	0.16	1.1

Continuous traffic	12-Jul-2007	3.8	1.1	33	0.07	0.03	0.78	0.01	0.02	0.10	0.02	0.24	0.13	4.9

Truck traffic	26-Jul-2007	2.1	2.3	43	0.04	0.03	0.90	0.01	0.02	0.04	0.01	0.04	0.13	8.1

Stop & Go traffic	24-Jan-2008	12	0.30	16	0.05	0.06	0.05	0.01	0.04	0.03	0.03	0.00	0.03	6.4

Underground	26-Sep-2007	2.3	27	305	0.27	0.13	12	0.00	0.00	0.00	0.00	0.09	0.03	29

Data of the urban background and farm site was not available due to equipment failure on the sampling days chosen for the in vitro toxicity testing. Values of trace metals represent the total of the soluble and insoluble metal fraction. PAH, polycyclic aromatic hydrocarbon, represents the total of 18 PAHs: naphthalene, acenaphthalene, acenaphthene, fluorine, phenanthrene, anthracene, fluoranthene, pyrene, benzo(a)anthracene, chrysene, benzo(b)fluoranthene, benzo(k)fluoranthene, benzo(e)pyrene, benzo(a)pyrene, indeno(1,2,3-cd)pyrene, dibenz(a, h)anthracene, benzo(ghi)perylene, and coronene. EC, elemental carbon; OC, organic carbon. - below detection limit

**Table 3:** Chemical composition of fine particulate matter (PM) collected at the selected sampling days

Fine PM		Trace metals						Soluble inorganic compounds				Organic compounds		
**(< 2.5 μm)**		**(ng/μg PM)**						**(μg/μg PM)**				**(μg/μg PM)**		**(pg/μg PM)**

**Site**	**Sampling day**	**Al**	**Cu**	**Fe**	**Ni**	**V**	**Zn**	**NH_4_^+^**	**Cl^-^**	**NO_3_^-^**	**SO_4_^2-^**	**EC**	**OC**	**PAH**

Steelworks	16-Jul-2007	2.0	-	15	0.39	1.3	7.3	0.17	0.01	0.13	0.32	0.12	0.15	133

Harbor	13-Dec-2007	0.56	0.13	4.1	0.22	0.58	2.2	0.17	0.01	0.37	0.17	0.09	0.06	93.7

Continuous traffic	12-Jul-2007	2.0	1.7	40	0.21	0.45	5.3	0.14	0.02	0.18	0.22	0.96	0.57	125

Truck traffic	26-Jul-2007	2.0	1.7	35	0.04	0.15	4.5	0.12	0.00	0.09	0.18	1.02	0.42	251

Stop & Go traffic	24-Jan-2008	21	0.15	19	0.33	0.59	2.3	0.04	0.03	0.04	0.12	0.19	0.07	231

Underground	26-Sep-2007	5.4	41	464	0.34	0.14	23	0.00	0.01	0.00	0.01	0.26	0.12	2.98

Data of the urban background and farm site was not available due to equipment failure on the sampling days chosen for the *in vitro *toxicity testing. Values of trace metals represent the total of the soluble and insoluble metal fraction. PAH, polycyclic aromatic hydrocarbon, represents the total of 18 PAHs: naphthalene, acenaphthalene, acenaphthene, fluorine, phenanthrene, anthracene, fluoranthene, pyrene, benzo(a)anthracene, chrysene, benzo(b)fluoranthene, benzo(k)fluoranthene, benzo(e)pyrene, benzo(a)pyrene, indeno(1,2,3-cd)pyrene, dibenz(a, h)anthracene, benzo(ghi)perylene, and coronene. EC, elemental carbon; OC, organic carbon. - below detection limit

### Oxidative potential

All the concentrated PM samples, regardless of site, showed oxidative potential as assessed in the DTT-assay (Additional file 2, table s1). For three sites, there was insufficient sample to test all PM size fractions (harbor coarse and qUF; steelworks qUF, continuous traffic qUF). DTT consumption was highest for the underground train station site in all size fractions (coarse 0.484, fine 0.617 and qUF 0.666 nmol DTT/μgPM*min), and lowest for qUF PM of the urban background site (0.010 nmol DTT/μgPM*min).

### Endotoxin content

Endotoxin was detectable in all the PM samples that were used for the *in vitro *exposures (Additional file 3 table s2; column "after concentration"). This is in agreement with previous reports indicating that endotoxin can be present in mostly the coarse, and also in the fine and ultrafine fractions collected with a BioSampler connected to a VACES [9,29]. However, the endotoxin content of five samples in the coarse size range, and one sample in the fine size range, was extremely high (> 1000 EU/mg PM). These six samples also induced a strong pro-inflammatory response *in vitro *(data not shown), and the observed minimal effect concentrations were very different from the other PM samples. Even at the lowest concentration tested, the six PM samples with high endotoxin content induced a stronger pro-inflammatory response than the highest concentration of all other PM samples.

We concluded that the endotoxin concentration in these six PM samples became artificially high during the sample processing in the laboratory (paragraph 2.2., concentrating the samples by evaporating the water). Bacteria present on particles surface could have multiplied during the sample concentration at 25°C. Additional measurements showed that the endotoxin levels were indeed 5.5-57 times lower before concentration (Additional file 3, table s2; column "before concentrating"). The factor increase was not equal for all samples, and independent of the duration of the concentration procedure (data not shown). This possibly indicates that the live bacterial load was different for each sample.

Because of the likelihood that new endotoxin was formed during the concentration process, and endotoxin is known to induce a pro-inflammatory response *in vitro *[15,16], we decided to exclude these six samples (samples with an extreme high endotoxin levels) from our data-analysis on the pro-inflammatory markers.

### In vitro toxicity

#### MTT-reduction activity

**Figure 1:**
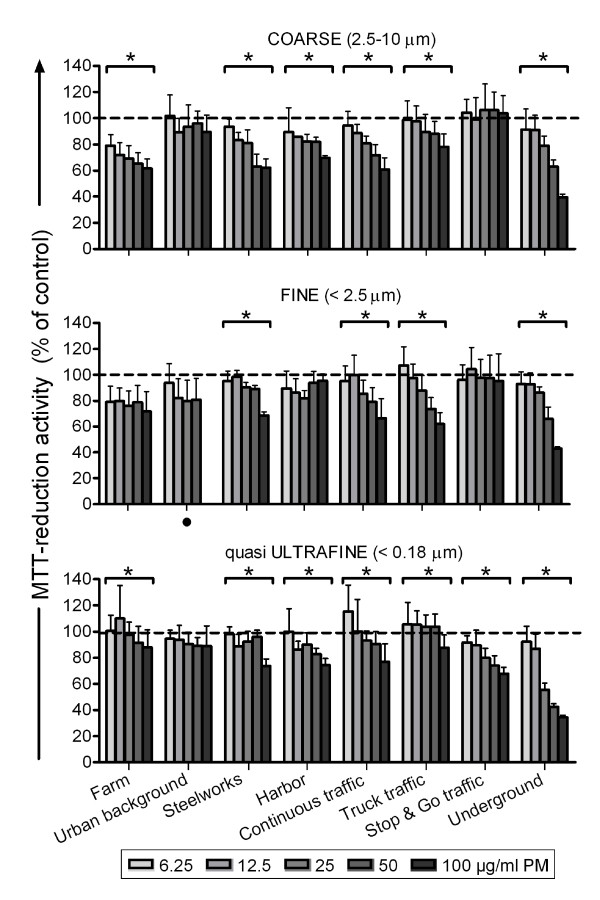
**MTT-reduction activity of RAW 264.7 macrophages after 16 hours of incubation with the PM samples**. Each bar shows mean ± standard deviation of two exposures in triplicate. Data was analyzed using multiple linear regression. * Statistically significant different from no concentration-response p < 0.05. · Highest concentration not tested (not enough sample).

Figure 1 shows the MTT-reduction activity of macrophages after 16 hours exposure to the concentrated PM samples. PM from most sites caused a concentration-dependent decrease in MTT-reduction activity. The underground site was by far the most potent whereas the urban background site did not cause a concentration-dependent decrease in all PM size fractions (Additional file 4, table s3). A relatively small decrease in MTT-reduction activity (< 20%) was observed after exposure to fine PM collected at the farm site, as well as fine and qUF PM collected at the urban background site. However, this was not concentration-dependent in the linear regression model and therefore not statistically significant. For some sites, there were statistically significant differences in MTT-reduction activity between the PM size fractions (Additional file 4, table s3). For example, coarse and fine PM from the stop & go traffic site did not affect MTT-reduction activity whereas qUF PM caused a concentration-dependent decrease. However, taken all sites together, the average MTT-reduction activity to the coarse, fine and qUF PM did not differ from each other. This result did not change following exclusion of the underground site.

#### Pro-inflammatory markers

**Figure 2:**
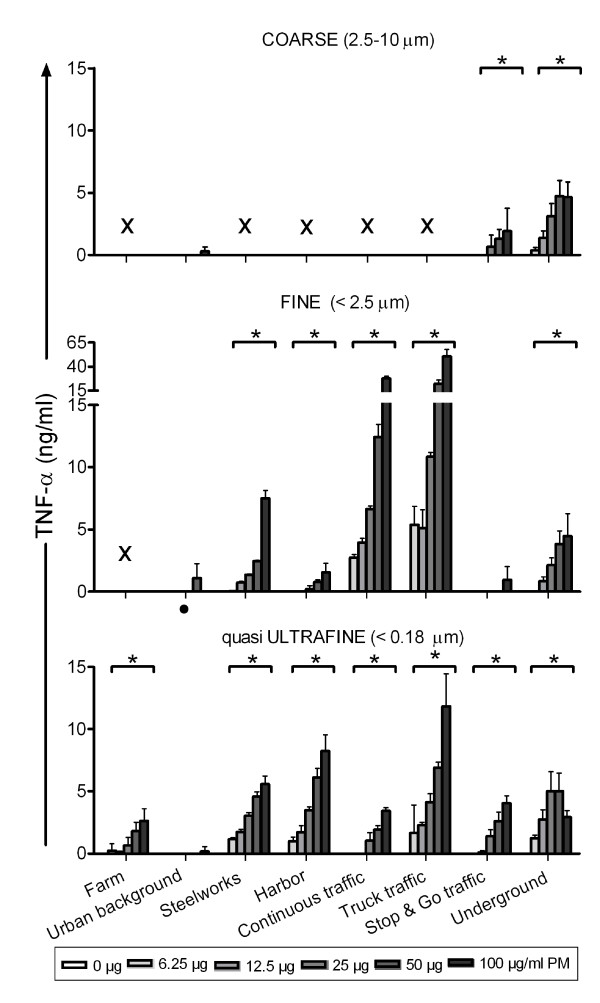
**TNF-α produced by RAW 264.7 macrophages after 16 hours incubation with the PM samples**. Each bar shows mean ± standard deviation of two exposures in triplicate. Data was analyzed using multiple linear regression. * Statistically significant different from no concentration-response p < 0.05. × Sample excluded because of high endotoxin level. · Highest concentration not tested (not enough sample).

**Figure 3:**
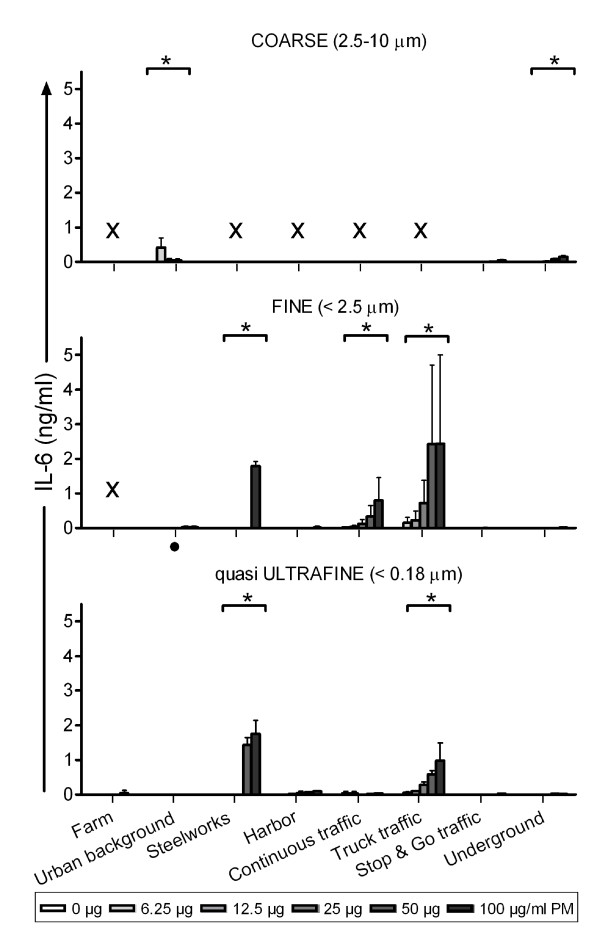
**IL-6 produced by RAW 264.7 macrophages after 16 hours incubation with the PM samples**. Each bar shows mean ± standard deviation of two exposures in triplicate. Data was analyzed using multiple linear regression. * Statistically significant different from no concentration-response p < 0.05. × Sample excluded because of high endotoxin level. · Highest concentration not tested (not enough sample).

**Figure 4:**
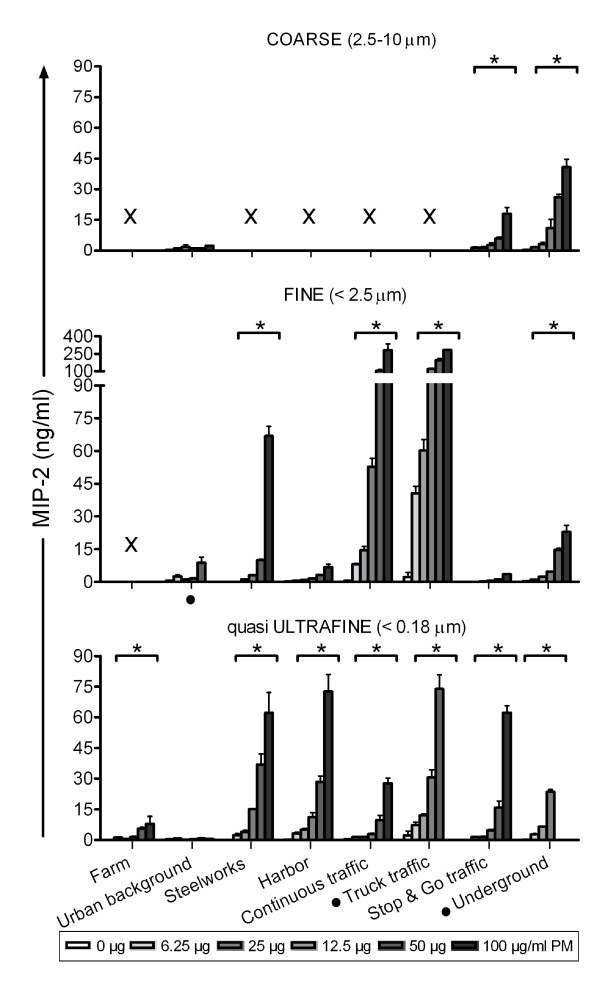
**MIP-2 produced by RAW 264.7 macrophages after 16 hours incubation with the PM samples**. Each bar shows mean ± standard deviation of one exposures in triplicate. Data was analyzed using multiple linear regression. * Statistically significant different from no concentration-response p < 0.05. × Sample excluded because of high endotoxin level. · Highest concentration not tested (not enough sample).

The production of pro-inflammatory cytokines TNF-α and IL-6, and chemokine MIP-2 were determined in the cell's supernatant after 16 hours of PM incubation (Figure 2, 3 and 4). The results are described by PM size fraction.

In the coarse fraction, five samples were excluded from data analysis because of high endotoxin levels leaving a total of three sites. Coarse PM collected at the underground train station site induced a statistically significant concentration-dependent increase in TNF-α, IL-6 and MIP-2 production. Similar effects were observed for PM from the stop & go traffic site. In contrast, levels of pro-inflammatory markers were low (IL-6) or not detectable (TNF-α and MIP-2) after exposure to urban background PM.

For the fine fraction, one sample (Farm site) was excluded from data analysis due to extremely high endotoxin levels. In general, fine PM caused a concentration-dependent increase in the production of pro-inflammatory markers, with exception of PM from the urban background and stop & go traffic site. The greatest increase in TNF-α and MIP-2 production was observed for the truck traffic site, followed by the continuous traffic and thereafter the steelworks site (Additional file 4, Table s3). Consistent with these data, the same three sites also induced the largest IL-6 response (truck traffic > steelworks > continuous traffic).

In the qUF size fraction, all samples induced a significant concentration-dependent increase in TNF-α and MIP-2 production, with exception of the urban background sample. For IL-6, only the steelworks and truck traffic samples induced a significant concentration-dependent increase. In this size range, the most marked increases in pro-inflammatory markers were observed following exposure to PM collected at the truck traffic, steelworks and harbor site (Additional file 4, table s3).

Overall, the qUF induced increase in pro-inflammatory markers was lower than in the fine size fraction (statistically significant lower for TNF-α and MIP-2 [p < 0.001 and p = 0.006 respectively], and nearly significant for IL-6 [p = 0.0531]). Excluding the underground site did not alter these results appreciably (Additional file 4, table s3). Size related differences including the coarse fraction could not be analyzed, since several coarse samples were excluded because of their high endotoxin content.

### Associations between endotoxin, oxidative potential and in vitro toxicity

There was no association between PM endotoxin content and the MTT-reduction activity of macrophages after exposure to PM (Additional file 5, table s4 and Additional file 6, figure s1A). However, there was a modest, statistically significant association between endotoxin and production of pro-inflammatory markers (Additional file 5, table s4 and Additional file 6, figure s1B-C). Excluding the underground train station site did not change this result for either MTT-reduction activity or production of pro-inflammatory markers.

**Figure 5:**
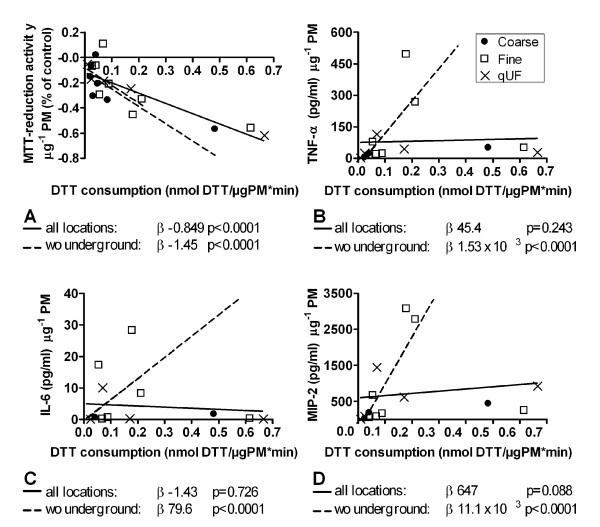
**Relationship between the particles' oxidative potential (DTT consumption) and cellular responses in RAW 264.7 macrophages**. Cells were exposed to increasing concentrations of PM after which MTT-reduction activity and the release of pro-inflammatory markers was measured. For each cellular response parameter, the slope of the concentration-response curve is plotted against the corresponding DTT consumption of each PM sample. The solid lines represent correlation investigated for all sites, dotted lines without the underground train station site (wo underground). Panel A: MTT-reduction activity (n = 20 PM samples; 8 sites × 3 PM size fractions per site minus 4 samples that were not tested in the DTT-assay since there was not enough material). Panel B-D: release of pro-inflammatory markers (n = 15 PM samples, 20 minus 5 more samples that were excluded because of high endotoxin levels). Statistical analysis was performed by multiple linear regression and shown as effect estimate (β, slope) and belonging p-value.

PM oxidative potential was significantly negatively associated with MTT-reduction activity whereas there was no association between oxidative potential and the production of pro-inflammatory markers (Figure 5, and Additional file 7, table s5). The latter appeared to be due to the underground train station site, since after excluding this site, there was a significant positive association between oxidative potential and each pro-inflammatory marker (Figure 5, and Additional file 7, table s5). Associations between oxidative potential and MTT-reduction activity or production of inflammatory markers were not influenced by PM endotoxin content as assessed in a two component regression model (data not shown).

## Discussion

In this study, we examined the *in vitro *pro-inflammatory and cytotoxic effects of PM of different sizes collected at different sites in the Netherlands. All samples, with exception of urban background and stop & go traffic PM, caused a concentration-dependent decrease in MTT-reduction activity and increase in production of TNF-α, IL-6 and MIP-2. The pro-inflammatory response was highest for fine PM collected at traffic locations whereas the MTT-reduction activity was most influenced by the underground train station site. Furthermore, MTT-reduction activity was negatively associated with PM oxidative potential for all PM size fractions. For pro-inflammatory response, a significant positive association with DTT consumption was only observed when only the outdoor sites were considered.

### Air pollution characteristics

Per site, the sampling day with highest PM concentration (μg/ml) was chosen for *in vitro *toxicity testing since there had to be sufficient sample for testing the full concentration range. Our results should therefore be interpreted with caution as these single day samples may not be representative for the sites on a yearly basis. One sample per site, collected at different days, rather reflects differences between sampling days. This implicates that the observed differences in *in vitro *toxicity between our PM samples can be related to differences in PM composition, but cannot be contributed to site specific characteristics (for example, stop & go traffic emissions are less toxic than truck traffic emissions).

In figure S2 (Additional file 8) the air pollution characteristics of the selected sampling days are compared to site averages of the full sampling campaign. The most striking difference between the site averages and selected samples was observed for the stop & go traffic location. At the selected sampling day for this site, median particle number concentration was substantially higher (64,700 particles/cm^3 ^compared to the site average of 53,000 particles/cm^3^). During this sampling day, PM_10 _concentrations were similarly elevated across the entire country, suggesting a regional pollution episode as the cause (data obtained from the Dutch National Air Quality Monitoring Network, not shown). Nonetheless, urban concentrations were well in excess of rural levels, and PM samples from this day represent local emissions imposed upon a substantial regional background.

### Chemical composition and in vitro toxicity

The *in vitro *toxicity differed between PM of different sizes and collection sites in the Netherlands. In all PM size ranges, underground PM clearly caused the largest decrease on MTT-reduction activity expressed per μg PM. The effect on MTT-reduction activity might be linked to the high metal content, which was substantially higher compared to the other sites. At the underground train site, the most abundant metal species were iron and copper. In coarse PM, the amount of iron per μg PM was 7 to 50 times higher and the amount of copper was 12 to 385 higher compared to the other sites (Table 2). Similar contrasts were observed for fine PM: 11 to 113 times more iron and 24-315 times more copper per μg PM (Table 3). Several other studies showed that particulate samples with high metal content evoked inflammatory responses as well as cytotoxic responses *in vitro *and *in vivo *[17,30-32]. For example, Gerlofs-Nijland *et al*. [17] compared the effects of PM samples collected in two cities that different in metal content in compromised rats. The results showed that both samples triggered inflammatory responses, which could be partially inhibited by addition metal chelator DTPA (diethylene triamine pentaacetic acid), but still the sample richer in metals revealed a greater enhancement in inflammatory responses. In addition, a recent *in vitro *study by Perrone *et al*. [32] revealed that the biological responses of a human lung epithelial cell line to urban fine PM were related to several (metal) elements: cell viability reduction was related with arsenic, zinc, chromium, copper and manganese; DNA damage with other elements like iron, chromium and cadmium (and possibly PAHs), and IL-8 production to arsenic, zinc and inorganic ions like sulfate.

In the fine size range, the pro-inflammatory activities (TNF-α and MIP-2 production) were highest for the truck traffic and continuous traffic site. This observation is in line with findings of Seagrave *et al*. [33], who exposed rats to fine PM collected at sites with different contributing PM sources. Seagrave *et al*. observed that the most toxic (/pro-inflammatory) samples were from the sites with the largest contributions of diesel and gasoline emissions. Our truck traffic and continuous traffic sites were similar in that they had the highest EC and OC content, which is characteristic (though not exclusive) for traffic emissions sources [34,35]. It has previously been reported that a high EC and OC content is associated with pro-inflammatory activity [36,37]. In most studies, the pro-inflammatory response was driven by compounds adsorbed at the carbon surface, such as transition metals or PAHs, rather than by EC or OC themselves [38-41].

Importantly, whereas fine PM from the continuous traffic and truck traffic sites did show pro-inflammatory activity, the sample from the other traffic related site, stop & go traffic, did not. In addition, this sample had a relatively low EC and OC content. This further strengthens the idea that the selected stop & go traffic sampling day was strongly influenced by other emissions sources, and thus did not represent average conditions at this site. In contrast, qUF PM from this site did induce a modest pro-inflammatory response (TNF-α and MIP-2). Since particles are known to grow in diameter as the distance from their source increases [42], the majority of qUF collected at this site therefore may still have originated from stop & go traffic.

Interestingly, the PM samples from the underground site caused the largest decrease in MTT-reduction activity, but not the highest pro-inflammatory activity. Similar effects were observed in another *in vitro *study by Karlsson *et al*. [43]. They compared the genotoxicity and the ability to induce inflammatory mediators of particles from different sources (wood combustion, tire-road wear, an urban street and a subway station). Karlsson *et al*. found that particles from subway were most genotoxic of all particles tested. However, particles from street level were the most potent to induce inflammatory cytokines. Furthermore, *in vitro *differences in toxicity versus pro-inflammatory activity were also observed by Gualtieri *et al*. [44]. They observed that urban PM samples collected in winter were more cytotoxic than summer samples, whereas the summer PM samples exhibited a higher pro-inflammatory potential. A possible explanation could be that the observed toxicity led to reduced responsiveness to pro-inflammatory triggers. Another possible explanation might be that different PM characteristics are responsible for inducing cytotoxicity and causing pro-inflammatory effects [6,44-46]. In the study of Jalava *et al*. [6] mouse RAW 264.7 macrophages were exposed to coarse, fine and qUF PM from six European urban background sites. Their data showed considerable heterogeneity between the PM samples with regard to both pro-inflammatory activity as well as cytotoxic activity, but there was no statically significant correlation between these parameters. In addition, Guastadisegni *et al*. [45] showed that the metal chelator DTPA inhibited arachidonic acid release in RAW 264.7 cells exposed to traffic-related PM, whereas recombinant endotoxin-neutralizing protein partially inhibited TNF-α production, demonstrating that different PM components triggered pro-inflammatory responses through separate pathways.

In the fine and qUF size range, there were a few PM samples causing a small, non-linear decrease in MTT-reduction activity (farm fine, urban background fine and qUF PM). The absence of a linear concentration-dependent effect does not necessarily imply that there is no effect at all. A possible explanation could be that these PM samples contain a compound with high "potency", but low "efficacy" to influence MTT-reduction activity. In that case, even the lowest concentration used for the in vitro exposures would be within the maximum effect range. Unfortunately, there is no chemical composition data available for the sampling days at the farm and urban background site. Therefore, it is not possible to relate these finding to specific components of ambient PM.

### Oxidative potential and in vitro toxicity

In all PM size ranges, the underground site had very high oxidative potential (Additional file 2, table s1). This might be very well caused by high metal levels, since those were much higher in the underground samples then for the other sites. In line with these findings, Ntziachristos *et al*. [47] found high correlations of transition metals and DTT consumption for PM_2.5 _and PM_0.15 _samples collected at different sites in California, USA. Furthermore, metals are known to catalyze the oxidation of DTT as measured by the DTT assay [48,49]. Li *et al*. [8] observed a significant correlation (r^2 ^0.98) between DTT consumption and PAH content of coarse and fine PM. Conversely, when we performed the same (statistical) analysis on our data, we found no significant association between PAH and DTT consumption (correlation < 0.25, p-value > 0.1; data not shown). However, in our study, PM chemical composition (including PAH) was determined on filter extracts, whereas (concentrated) impinger samples were used for the *in vitro *studies. Analysis of the association between PAH and DTT was thus not performed on the same type of samples. Also, the number of samples available for this analysis was limited (n = 11, Table 2 Table 3 and Additional file 2, table s1), since we have examined only one sampling day per site. An in-depth analysis on associations between oxidative potential and PM composition (including all sampling days for each site) will be published elsewhere (Godri *et al*., manuscript in preparation).

A more general approach is to correlate DTT consumption to the observed *in vitro *toxicity. In our study, PM samples that had high DTT consumption caused a decrease in MTT-reduction activity. This association was statistically significant even after excluding the underground sample (highest data point for both decreases in MTT-reduction activity as well as oxidative potential). In addition, samples with a high DTT consumption also had a high pro-inflammatory activity except for the underground sample. As mentioned above, this might be attributable to the fact that different elements may activate different cellular responses or that the observed toxicity hampers the cellular responsiveness.

To date, several other in vitro studies have been published that used the DTT assay to determine the oxidative potential of (ambient) PM [21,50-52]. Most of these studies focused on the relationship between chemical composition and DTT-activity of PM. So far, limited research is performed on the association between the in vitro DTT-activity and the in vitro or in vivo toxicity of PM. For example, Li *et al*. [8] demonstrated that cellular heme oxygenase 1 (HO-1, an enzyme responsive to oxidative stress) expression was correlated to DTT-activity. However, to our best knowledge, there is currently no clear relationship with adverse health effects in humans.

### PM size fraction related effects

In the present study, PM size fraction did not seem the principle determinant of MTT-reduction activity. Although there were significant differences between the coarse, fine and qUF fractions within three sites (harbor, truck traffic and stop & go traffic), these were not pointing towards one direction (e.g. decrease in MTT-reduction activity). Considering all sites together, there were no statistically significant differences between the size fractions (Additional file 4, table s3). The observation that PM size fraction is not the most important PM characteristic in determining PM effect on MTT-reduction activity has been previously reported [6,53].

In contrast, the pro-inflammatory activity of the fine size fraction was significantly higher than the qUF size fraction. Numerous studies have attempted to link PM size to pro-inflammatory activity [5,54-56]. For example, Hetland *et al*. [56] showed that coarse PM was more potent at inducing induce pro-inflammatory cytokines than fine PM *in vitro*, whereas de Haar *et al*. [54] showed that ultrafine but not fine particulate matter causes airway inflammation *in vivo*. However, several recent (*in vitro*) studies have shown that both PM size as well as PM composition contributed to adverse effects of PM exposure [57,58]. Ramgolam *et al*. [57]exposed human airway epithelial cells to size-fractionated PM collected at an urban background site in Paris. They used isomass exposures (same particle concentrations for each size fractions) as well as isovolume exposures (same volume of particles in suspension; to respect the particle proportions observed in ambient air) and characterized the pro-inflammatory response by measuring the pro-inflammatory cytokine Granulocyte Macrophage-Colony Stimulating Factor (GM-CSF). Ramgolam *et al*. found that the pro-inflammatory response decreased with aerosol size when cells were exposed to isomass of particle suspension. However, when cells were exposed to isovolume of particle suspensions, the GM-CSF release was maximal with the fine fraction. The authors concluded that this could be related to the chemical composition of each size fraction. In another *in vitro *study, by Osornio-Varga et al [58], urban PM_10 _and PM_2.5 _was investigated in three different biological systems. For most, but not all, parameters they found a significant effect of PM size: hemolysis and induction of DNA degradation were pre-dominantly induced by PM_10 _whereas the inhibition of cell proliferation was significantly stronger by PM_2.5 _(not size dependent: TNF-α and IL-6 release). In addition, results of PM elemental composition principal component analysis were used in associating cellular effects. This revealed that different elements triggered specific biological parameters. Taken together, it is most likely that the combination of PM characteristics, such as source-dependent PM composition and oxidative potential, determines the toxicity rather than a single PM characteristic as PM size. In this study, the observed differences in pro-inflammatory response after in vitro exposures of fine and qUF PM was likely caused by the differences in chemical composition rather than the particle size per se.

### Limitations of this study

The PM samples used for *in vitro *toxicity testing were collected using a liquid impinger system (VACES), whereas ambient PM is usually collected on filters. Impingers have the advantage over filter-based systems that PM does not have to be extracted from the filter before use, and might therefore better represent ambient particle characteristics. However, HVS filter extracts were used to determine PM chemical composition. This hampers the interpretation of the chemical composition data in relation to the observed *in vitro *toxicity.

In addition, despite the enrichment of the aerosols using a VACES, insufficient mass was collected to perform toxicity studies at the desired concentrations and PM samples thus had to be concentrated in the laboratory. We tried to avoid changing particle properties by using a mild concentration procedure. Therefore, we concentrated the samples by evaporating the water at 25°C under a nitrogen flow to avoid biological contamination and avoid heating samples to a high temperature or freeze-drying. Nevertheless, the endotoxin content of six concentrated samples turned out to be extremely high. A possible explanation could be that biological material present in the sample multiplied during the concentration procedure. Additional measurements in the corresponding un-concentrated samples showed that the endotoxin levels were indeed 5.5-57 times lower before concentration (Additional file 3, table s2). Consequently, the endotoxin levels in the concentrated samples as used for the *in vitro *exposures most likely do not reflect ambient endotoxin levels.

However, although the endotoxin levels in our concentrated impinger samples may not reflect ambient endotoxin levels, the biological material most likely originates from the ambient PM itself, and not from laboratory contamination. Firstly, because endotoxin in ambient PM is associated with the coarse fraction [59] and five out of the six outliers in the present study were coarse samples. The sixth sample containing high endotoxin levels was a fine sample from the farm site were endotoxin levels were expected to be high. Second, the endotoxin levels in our un-concentrated samples are within the same range as measured by others who used VACES to collected ambient PM [9,29]. For example, the endotoxin concentration PM collected in Mexico City ranged from 16 to 895 EU per mg PM [29]. Finally, most importantly, because we have also measured endotoxin in PM_10 _filter extracts. This data is available for all RAPTES sampling days and is recently published by Strak *et al*. [23]. The average PM_10 _endotoxin concentration at our sampling sites (Strak *et al*., Table 2: farm 26.15 EU/m^3^, other sites ≤ 1.71 EU/m^3^) are in the same order of magnitude as observed by others at outdoor sites [12,60,61]. For future *in vitro *or *in vivo *exposure studies with either impinger or filter extracts, we recommend to treat PM samples in such way that endotoxin effects can be either avoided or assessed in a controlled manner. For example, to irradiate samples before use or to combine PM exposures with an endotoxin blocker/inhibitor such as polymyxin or recombinant endotoxin-neutralizing protein (rENP).

Another point of consideration is that all the comparisons between toxic activities of the PM samples used in this study have been made on mass basis. This does not take into account the exposure levels at each site. For example, PM from the steel work site had relatively high pro-inflammatory activity, but the ambient PM concentration at this site was relatively low compared to the other sites. In other words, although the hazard for induction of inflammation by the steelworks site would be large, the risk would be small since the PM exposure at this site is low. In addition, the deposited dose in the lung and respiratory tract depends on the aerodynamic size of PM, but this is not included in this *in vitro *study. For risk management, not only the potency but also the exposure and dose have to be taken into consideration.

## Conclusion

In summary, the pro-inflammatory response was highest for PM collected at traffic locations (marked by their high EC and OC concentrations) whereas the largest decrease in MTT-reduction activity was caused by underground train station PM (high in metal components). However, the relative small sample size of the present study does not allow the drawing of firm conclusions on associations between *in vitro *toxicity and specific PM components. In this study, PM size fraction was not related to MTT-reduction activity, whereas there was a statistically significant difference in pro-inflammatory activity between Fine and qUF PM. Furthermore, PM oxidative potential was significantly negatively associated with MTT-reduction activity. In conclusion, the response of RAW264.7 cells to ambient PM was markedly different using samples collected at various sites in the Netherlands that differed in their local PM emission sources. Our results are in support of other investigations showing that the chemical composition as well as oxidative potential are determinants of PM induced toxicity *in vitro*.

## Abbreviations

CPC: Condensation Particle Counter; CV: Coefficient of variation; Dp: Aerodynamic diameter; DFO: Desferrioxamine; DTPA: Diethylene Triamine Pentaacetic Acid; DTT: Dithiothreitol; EC: Elemental Carbon; ELISA: Enzyme-Linked Immunosorbent Assay; GM-CSF: Granulocyte Macrophage-Colony Stimulating Factor; HVS: High-Volume Sampler; IL-6: Interleukin-6; LAL assay: Limulus Amoebocyte Lysate assay; LPS: Lipopolysaccharide; MIP-2: Macrophage Inflammatory Protein-2; MOUDI: Micro-Orifice Uniform Deposit Impactor; MTT: 3-(4,5-dimethylthiazol: 2-yl)-2,5-diphenyl-tetrazolium bromide; OC: Organic Carbon; PAHs: Polycyclic Aromatic Hydrocarbons; PM: Particulate Matter; PNC: Particle Number Concentration; qUF: quasi ultrafine size fraction (< 0.18 μm); RAPTES: Risk of Airborne Particles: a hybrid Toxicological and Epidemiological Study; rENP: recombinant endotoxin-neutralizing protein (rENP); ROS-Reactive Oxygen Species; TNF-α: Tumor Necrosis Factor-alpha; VACES: Versatile Aerosol Concentration Enrichment System

## Competing interests

The authors declare that they have no competing interests.

## Authors' contributions

MS and IG designed the *in vitro *study. MS and MMS collected the particulate matter. MMS analyzed the data on air pollution characteristics and meteorological conditions. KJG analyzed the chemical composition. MS carried out the *in vitro *study, analyzed and interpreted the data and drafted the manuscript. NAHJ and GH contributed to the statistical data analysis. RHHP participated in the design of the study and critically read the drafted manuscript. EL, FRC, NAHJ, BB, GH, RMH, FJK, and ISM designed the RAPTES project, and helped to draft manuscript. All authors critically read and approved the final manuscript.

## Supplementary Material

Additional file 1 **Additional information Methods**. Description of the DTT assay, cell culture conditions and MTT-assay. Click here for file

Additional file 2 **Table s1. Oxidative potential of particulate matter (PM) samples collected at eight contrasting sites measured by the DTT-assay**. DTT consumption was calculated based on linear regression on four data points (t0, t15, t30 and t45 minutes incubation time) and each data point represents the average of a duplicate measurement. NA, data not available. Click here for file

Additional file 3 **Table s2. Endotoxin levels measured in particulate matter (PM) samples collected at contrasting sites before and after concentration for *in vitro *toxicity testing**. Endotoxin content was determined using the LAL assay. To enable *in vitro *toxicity testing on equal PM mass concentration, all samples were concentrated by evaporation of water in PM (except for the underground railway station samples). Samples used for *in vitro *toxicity testing are depicted in bold. Values > 1000 EU/mg were above detection limit. Coarse PM (2.5-10 μm); fine PM (< 2.5 μm); qUF, quasi ultrafine PM (< 0.18 μm). NA, not available. Click here for file

Additional file 4 **Table s3. PM size fraction related differences in cellular responses of RAW 264.7 macrophages exposed to PM collected at eight contrasting sites**. Cells were exposed to increasing concentrations of particulate matter (PM) after which MTT-reduction activity and the release of pro-inflammatory markers was measured. Each PM sample was tested in triplicate in two independent experiments. Data are shown as slope ± standard error (SAS, multiple linear regression). Bold and bold+italic values were statistically significant different from the fine or coarse size fraction respectively (p < 0.05). The coarse fraction was not included in the data analysis on the pro-inflammatory markers (TNF-α, IL-6 and MIP-2), since too many samples had to be excluded because of high endotoxin values. C, coarse (2.5-10 μm); F, fine (< 2.5 μm); qUF, quasi ultrafine (< 0.18 μm); FA, Farm; UB, urban background; SW, steelworks; HA, harbor; CT continuous traffic; TT, truck traffic; SG, stop & go traffic; UN, underground railway station; n = number of sites; × = Excluded from data analysis because of high endotoxin levels. Click here for file

Additional file 5 **Table s4. Relationship between the PM endotoxin content and cellular responses in RAW 264.7 macrophages exposed to PM collected at eight contrasting sites**. Cells were exposed to increasing concentrations of PM after which MTT-reduction activity and the release of pro-inflammatory markers was measured. For each cellular parameter separately, the slope of the concentration-response curve was plotted against the corresponding endotoxin content of each PM sample. Subsequently, multiple linear regression was used to calculate the associations (β, slope, and belonging p-value) between endotoxin content and cellular responses. Six samples were excluded from data analysis with regard to the pro-inflammatory responses because of high endotoxin levels (5 coarse and 1 fine sample). The coarse fraction was not included in the data analysis on the pro-inflammatory markers (TNF-α, IL-6 and MIP-2), since n ≤ 3. n = number of sites included. wo underground, without underground railway station site. Bold values indicate statistically significant associations (p < 0.05). Click here for file

Additional file 6 **Figure s1. Relationship between the particulate matter (PM) endotoxin content and cellular responses in RAW 264.7 macrophages**. Cells were exposed to increasing concentrations of PM after which MTT-reduction activity and the release of pro-inflammatory markers was measured. For each cellular parameter, the slope of the concentration-response curve is plotted against the corresponding endotoxin content of each PM sample. The solid lines represent correlation investigated for all sites, dotted lines without the underground railway station site (wo underground). Panel A: MTT- activity (n = 24 PM samples; 8 sites × 3 PM size fractions). Panel B-D: release of pro-inflammatory markers (n = 18 PM samples, since 6 were excluded because of high endotoxin levels). Statistical analysis was performed by multiple linear regression and shown as effect estimate (β, slope) and belonging p-value. Click here for file

Additional file 7 **Table s5. Relationship between the PM oxidative potential (DTT consumption) and cellular responses in RAW 264.7 macrophages exposed to PM collected at eight contrasting sites**. Cells were exposed to increasing concentrations of particulate matter (PM) after which MTT-reduction activity and the release of pro-inflammatory markers was measured. For each cellular parameter separately, the slope of the concentration-response curve was plotted against the corresponding DTT consumption of each PM sample. Subsequently, multiple linear regression was used to calculate the associations (β, slope, and belonging p-value) between DTT consumption and cellular responses. Data on DTT consumption was missing for four samples (1 coarse and 3 qUF). Furthermore, six PM samples were excluded from data analysis with regard to the pro-inflammatory responses because of high endotoxin levels (5 coarse and 1 fine sample; one of the coarse samples was also lacking information on DTT consumption). The coarse fraction was not included in the data analysis on the pro-inflammatory markers (TNF-α, IL-6 and MIP-2), since n ≤ 3. n = number of sites included. wo underground, without underground railway station site. Bold values indicate statistically significant associations (p < 0.05). Click here for file

Additional file 8 **Figure s2. Mean values of air pollutants and meteorological conditions of the selected sampling day (red crosses) compared to the site average (black squares with standard deviation bars)**. PM sampling was carried out at eight sites for 4 - 12 separate days and for 6 hours per day at each site. Per site, the sampling day with highest PM concentration was chosen for *in vitro *toxicity testing to ensure sufficient availability of material for the various tests (selected days had highest coarse, fine and quasi UF particle concentrations, data not shown). Sampling at the farm, urban background and underground sites was independent from wind direction. PNC values were not available for the selected days of the steelworks and continuous traffic site. Background levels of PM_10 _were obtained from the Dutch National Air Quality Monitoring Network. PNC, particle number concentration (median). Click here for file
