# Supplementary material for: In vitro toxicity of particulate matter (PM) collected at different sites in the Netherlands is associated with PM composition, size fraction and oxidative potential - the RAPTES project
Source: Part Fibre Toxicol. 2011 Sep 2;8:26. doi: 10.1186/1743-8977-8-26 (PMC3180259; doi:10.1186/1743-8977-8-26)
Supplement: Additional file 1 — Additional information Methods. Description of the DTT assay, cell culture conditions and MTT-assay. [file 1743-8977-8-26-S1.PDF]

Additional information Methods

PM characterisation - DTT assay

This assay is based on the ability of redox active compounds associated with PM to transfer electrons from dithiothreitol (DTT), to oxygen. PM samples were incubated at 37 °C with DTT (100 µM) in 0.1 M potassium phosphate buffer at pH 7.4 (1 mL total volume) for increasing lengths of time (0, 15, 30 and 45 minutes). Each incubation was done in duplicate. The reaction was quenched by addition of 10% trichloroacetic acid, and aliquots of the quenched mixture were then transferred to a tube containing Tris HCl (0.4M Tris HCL, pH 8.9 in 20 mM EDTA), and 5,5′-dithiobis-2-nitrobenzoic acid (DTNB, 0.25 mM). The subsequent loss of DTT is followed by its reaction with DTNB to form 2-nitro-5-mercaptobenzoic acid, which is monitored spectrophotometrically at 415nm. The DTT consumed is determined from the difference between the mercaptobenzoate formed by the blank and that formed by the sample. The data collected at the multiple time points are used to determine the rate of DTT consumption which is normalized to the quantity of PM used in the incubation mixture [1].

The criteria for data reporting on a given sample were DTT consumption less than 25% and a linear consumption rate ($R^2 > 0.90$ for both samples). Furthermore, DTT consumption by the positive control should be +/- 0.05 nmol DTT/µgPM x min of the average of previously performed DTT assays (average of ten most recently performed assays). In addition, the DTT consumption of a given sample should be between 95 and 100% of the negative control at T=0. Samples that did not meet the reporting criteria were retested using higher or lower concentrations and shorter or longer time

points. Single or duplicate samples with a DTT consumption smaller than 0.007 nmol DTT/µgPM x min were reported as below detection limit.

In vitro exposure - cell culture

Cells were cultured and maintained in 75 cm^2 culture flasks (Greiner Bio-One, Solingen, Germany) in complete DMEM/F12 with glutamax I (GIBCO Invitrogen, Breda, The Netherlands) supplemented with 10% FCS (Greiner Bio-One, Solingen, Germany), 1% sodium pyruvate (GIBCO Invitrogen, Breda, The Netherlands) and 1% penicilline-streptomycine (GIBCO Invitrogen, Breda, The Netherlands). The cells were seeded $1.0*10^6$ cells per flask and cultured at 37°C in 5% CO_2. Cells grown to 80% confluency were harvested by scraping and subcultured in a new flask or used for the experiments.). The cells used in the experiments were between passages 9 and 30.

In vitro exposure - MTT assay

The MTT assay was performed as described by Mosmann [2]. The assay relies on the ability of the mitochondria to convert 3-(4,5-dimethylthiazol- 2-yl)-2,5-diphenyl-tetrazolium bromide (MTT) into an water-insoluble purple formazan. It evaluates the mitochondrial dehydrogenase activity and depends on the degree of cell activation [3]. In short, 3 mg/ml MTT (Sigma-Aldrich Steinheim, Germany) was prepared in phosphate buffered saline (PBS) and diluted five times in culture medium before each use. After overnight exposure to PM the cell culture medium was removed and 125 µl MTT solution was added to each well. Following 1 hour incubation at 37°C in 5% CO_2, the MTT solution was removed and 150µl isopropanol (Sigma-Aldrich, Steinheim, Germany) was added. The plates were gently shaken for 30 minutes in the

dark to dissolve the dark blue formazan crystals. 100 µl of the supernatant was brought into a clean 96-wells plate, and measured at 595 nm. MTT-reduction activity was expressed as percentage formazan formed by the exposed cells compared to the formazan formed by the unexposed cells.

References

1. Geller MD, Ntziachristos L, Mamakos A, Samaras Z, Schmitz DA, Froines JR, Sioutas C: **Physicochemical and redox characteristics of particulate matter (PM) emitted from gasoline and diesel passenger cars.** *Atmos Environ* 2006, **40:**6988-7004.

2. Mosmann T: **Rapid colorimetric assay for cellular growth and survival: application to proliferation and cytotoxicity assays.** *J Immunol Methods* 1983, **65:**55-63.

3. Gerlier D, Thomasset N: **Use of MTT colorimetric assay to measure cell activation.** *J Immunol Methods* 1986, **94:**57-63.
