# Supplementary material for: In vitro toxicity of particulate matter (PM) collected at different sites in the Netherlands is associated with PM composition, size fraction and oxidative potential - the RAPTES project
Source: Part Fibre Toxicol. 2011 Sep 2;8:26. doi: 10.1186/1743-8977-8-26 (PMC3180259; doi:10.1186/1743-8977-8-26)
Supplement: Additional file 2 — Table s1. Oxidative potential of particulate matter (PM) samples collected at eight contrasting sites measured by the DTT-assay. DTT consumption was calculated based on linear regression on four data points (t0, t15, t30 and t45 minutes incubation time) and each data point represents the average of a duplicate measurement. NA, data not available. [file 1743-8977-8-26-S2.PDF]

	DTT consumption (nmol DTT/µgPM x min)		
**Site**	**Coarse PM (2.5-10 µm)**	**Fine PM (< 2.5 µm)**	**qUF PM (< 0.18 µm)**
Farm	0.022	0.030	0.025
Urban background	0.026	0.091	0.010
Steelworks	0.032	0.057	NA
Harbor	NA	0.070	NA
Continuous traffic	0.086	0.214	NA
Truck traffic	0.049	0.180	0.070
Stop & Go traffic	0.043	0.043	0.171
Underground	0.484	0.617	0.666
