# Supplementary material for: In vitro toxicity of particulate matter (PM) collected at different sites in the Netherlands is associated with PM composition, size fraction and oxidative potential - the RAPTES project
Source: Part Fibre Toxicol. 2011 Sep 2;8:26. doi: 10.1186/1743-8977-8-26 (PMC3180259; doi:10.1186/1743-8977-8-26)
Supplement: Additional file 3 — Table s2. Endotoxin levels measured in particulate matter (PM) samples collected at contrasting sites before and after concentration for in vitro toxicity testing. Endotoxin content was determined using the LAL assay. To enable in vitro toxicity testing on equal PM mass concentration, all samples were concentrated by evaporation of water in PM (except for the underground railway station samples). Samples used for in vitro toxicity testing are depicted in bold. Values > 1000 EU/mg were above detection limit. Coarse PM (2.5-10 μm); fine PM (< 2.5 μm); qUF, quasi ultrafine PM (< 0.18 μm). NA, not available. [file 1743-8977-8-26-S3.PDF]

Site	Date	Fraction	EU/mg PM Before	EU/mg PM After	Factor increase
Farm	5-Jul-07	coarse	183	**> 1000**	> 5.5
		fine	35	**> 1000**	> 29
		qUF	13	**1.7**	0.13
Urban background	2-Jul-07	coarse	33	**8.9**	0.27
		fine	NA	**12**	-
		qUF	NA	**1.6**	-
Steelworks	16-Jul-07	coarse	19	**> 1000**	> 54
		fine	20	**15**	0.79
		qUF	NA	**1.8**	-
Harbor	13-Dec-07	coarse	NA	**> 1000**	-
		fine	NA	**1.5**	-
		qUF	NA	**1.8**	-
Continuous traffic	12-Jul-07	coarse	18	**> 1000**	> 57
		fine	NA	**3.6**	-
		qUF	NA	**0.91**	-
Truck traffic	26-Jul-07	coarse	55	**> 1000**	> 18
		fine	NA	**11**	-
		qUF	NA	**13**	-
Stop & Go traffic	24-Jan-08	coarse	10	**6.6**	0.65
		fine	4.2	**0.47**	0.11
		qUF	NA	**2.1**	-
Underground	26-Sep-07	coarse	**1.9**	NA	-
		fine	**0.76**	NA	-
		qUF	**9.3**	NA	-
