# Supplementary material for: In vitro toxicity of particulate matter (PM) collected at different sites in the Netherlands is associated with PM composition, size fraction and oxidative potential - the RAPTES project
Source: Part Fibre Toxicol. 2011 Sep 2;8:26. doi: 10.1186/1743-8977-8-26 (PMC3180259; doi:10.1186/1743-8977-8-26)
Supplement: Additional file 4 — Table s3. PM size fraction related differences in cellular responses of RAW 264.7 macrophages exposed to PM collected at eight contrasting sites. Cells were exposed to increasing concentrations of particulate matter (PM) after which MTT-reduction activity and the release of pro-inflammatory markers was measured. Each PM sample was tested in triplicate in two independent experiments. Data are shown as slope ± standard error (SAS, multiple linear regression). Bold and bold+italic values were statistically significant different from the fine or coarse size fraction respectively (p < 0.05). The coarse fraction was not included in the data analysis on the pro-inflammatory markers (TNF-α, IL-6 and MIP-2), since too many samples had to be excluded because of high endotoxin values. C, coarse (2.5-10 μm); F, fine (< 2.5 μm); qUF, quasi ultrafine (< 0.18 μm); FA, Farm; UB, urban background; SW, steelworks; HA, harbor; CT continuous traffic; TT, truck traffic; SG, stop & go traffic; UN, underground railway station; n = number of sites; × = Excluded from data analysis because of high endotoxin levels. [file 1743-8977-8-26-S4.PDF]

	**MTT-reduction activity (%) μg^{-1} PM**			**TNF-α (pg/ml) μg^{-1} PM**			**IL-6 (pg/ml) μg^{-1} PM**			**MIP-2 (pg/ml) μg^{-1} PM**		
	**C**	**F**	**qUF**	**C**	**F**	**qUF**	**C**	**F**	**qUF**	**C**	**F**	**qUF**
All sites	-0.222 ± 0.034 (n=8)	-0.222 ± 0.035 (n=8)	-0.238 ± 0.034 (n=8)	23 ± 15 (n=3)	1.5 x 10^2 ± 11 (n=7)	**46 ± 9.4 (n=8)**	0.13 ± 1.5 (n=3)	7.5 ± 1.1 (n=7)	4.0 ± 1.4 (n=8)	205 ± 138 (n=3)	779 ± 101 (n=7)	**398 ± 94.9 (n=8)**
All sites wo UN	-0.162 ± 0.037 (n=7)	-0.157 ± 0.037 (n=7)	-0.202 ± 0.037 (n=7)	12 ± 19 (n=2)	1.7 x 10^2 ± 12 (n=6)	**49 ± 11 (n=7)**	-0.62 ± 2.0 (n=2)	9.2 ± 1.3 (n=6)	**4.2 ± 1.6 (n=7)**	93.8 ± 178 (n=2)	911 ± 117 (n=6)	**400 ± 101 (n=7)**
By site												
FA	-0.155 ± 0.059	-0.074 ± 0.062	-0.170 ± 0.065	x	x	24 ± 4.8	x	x	0.0 ± 1.0	x	x	81.9 ± 34.6
UB	-0.068 ± 0.059	-0.213 ± 0.141	-0.055 ± 0.065	2.8 ± 4.2	20 ± 16	**1.5 ± 4.8**	-1.8 ± 0.88	0.44 ± 5.8	-0.01 ± 1.0	12.4 ± 14.7	152 ± 188	**1.85 ± 34.6**
SW	-0.309 ± 0.084	-0.298 ± 0.088	-0.206 ± 0.092	x	75 ± 11	**52 ± 6.8**	x	17 ± 3.9	20 ± 1.0	x	660 ± 88.6	654 ± 34.6
HA	-0.189 ± 0.084	***0.105 ± 0.062***	**-0.218 ± 0.065**	x	17 ± 7.8	**80 ± 5.0**	x	0.02 ± 2.8	**0.77 ± 1.0**	x	65.5 ± 88.6	**729 ± 34.6**
CT	-0.340 ± 0.065	-0.335 ± 0.066	-0.331 ± 0.065	x	27 x 10^2 ± 7.95	**36 ± 4.8**	x	8.1 ± 2.9	**0.11 ± 1.0**	x	2.77 x 10^3 ± 101	**275 ± 34.6**
TT	-0.210 ± 0.059	***-0.459 ± 0.066***	**-0.186 ± 0.072**	x	4.9 x 10^2 ± 7.95	**1.1 x 10^2 ± 5.2**	x	28 ± 2.9	10 ± 1.1	x	3.07 x 10^3 ± 124	**1.45 x 10^3 ± 85.9**
SG	0.018 ± 0.059	-0.066 ± 0.062	***-0.248 ± 0.065***	21 ± 4.2	9.2 ± 7.5	**44 ± 4.8**	0.52 ± 0.88	-0.01 ± 2.7	0.18 ± 1.0	1.8 x 10^2 ± 15	35.9 ± 88.6	**622 ± 34.6**
UN	-0.571 ± 0.059	-0.563 ± 0.062	-0.619 ± 0.077	49 ± 4.6	48 ± 8.1	28 ± 5.6	1.6 ± 0.88	0.15 ± 2.7	0.18 ± 1.4	4.3 x 10^2 ± 15	241 ± 88.6	**924 ± 179**
