# Supplementary material for: In vitro toxicity of particulate matter (PM) collected at different sites in the Netherlands is associated with PM composition, size fraction and oxidative potential - the RAPTES project
Source: Part Fibre Toxicol. 2011 Sep 2;8:26. doi: 10.1186/1743-8977-8-26 (PMC3180259; doi:10.1186/1743-8977-8-26)
Supplement: Additional file 5 — Table s4. Relationship between the PM endotoxin content and cellular responses in RAW 264.7 macrophages exposed to PM collected at eight contrasting sites. Cells were exposed to increasing concentrations of PM after which MTT-reduction activity and the release of pro-inflammatory markers was measured. For each cellular parameter separately, the slope of the concentration-response curve was plotted against the corresponding endotoxin content of each PM sample. Subsequently, multiple linear regression was used to calculate the associations (β, slope, and belonging p-value) between endotoxin content and cellular responses. Six samples were excluded from data analysis with regard to the pro-inflammatory responses because of high endotoxin levels (5 coarse and 1 fine sample). The coarse fraction was not included in the data analysis on the pro-inflammatory markers (TNF-α, IL-6 and MIP-2), since n ≤ 3. n = number of sites included. wo underground, without underground railway station site. Bold values indicate statistically significant associations (p < 0.05). [file 1743-8977-8-26-S5.PDF]

	**MTT-reduction activity (%) EU/mg PM $^{-1}$ (p-value) n**	**TNF-α (pg/ml) EU/mg PM $^{-1}$ (p-value) n**	**IL-6 (pg/ml) EU/mg PM $^{-1}$ (p-value) n**	**MIP-2 (pg/ml) EU/mg PM^{-1} (p-value) n**
All size fractions				
all sites	9.8 x 10^{-7} (0.1559) n=24	**10 (<.0001) n=18**	**1.0 (<.0001) n=18**	**39 (0.0062) n=18**
all sites wo underground	3.8 x 10^{-7} (0.5384) n=21	12 (<.0001) n=15	1.1 (<.0001) n=15	37 (0.0227) n=15
By size fraction				
coarse	6.3 x 10^{-7} (0.4053) n=8	n=3	n=3	n=3
coarse wo underground	1.1 x 10^{-7} (0.9880) n=7	n=2	n=2	n=2
fine	3.8 x 10^{-6} (0.0656) n=8	**18 (<.0001) n=7**	**1.7 (<.0001) n=7**	**51 (0.0482) n=7**
fine wo underground	2.1 x 10^{-6} (0.2974) n=7	**18 (<.0001) n=6**	**1.7 (<.0001) n=6**	40 (0.1810) n=6
qUF	-0.011 (0.2073) n=8	**4.7 (<.0001) n=8**	0.42 (0.0684) n=8	**1.1 x 10^{2} (<.0001) n=8**
qUF wo underground	0.002 (0.7398) n=7	**6.9 (<.0001) n=7**	**0.63 (0.0171) n=7**	**1.1 x 10^{2} (<.0001) n=7**
