# Supplementary figures and images for: In vitro toxicity of particulate matter (PM) collected at different sites in the Netherlands is associated with PM composition, size fraction and oxidative potential - the RAPTES project
Source: Part Fibre Toxicol. 2011 Sep 2;8:26. doi: 10.1186/1743-8977-8-26 (PMC3180259; doi:10.1186/1743-8977-8-26)

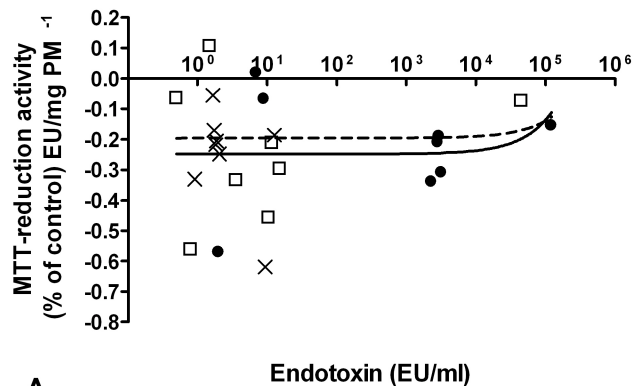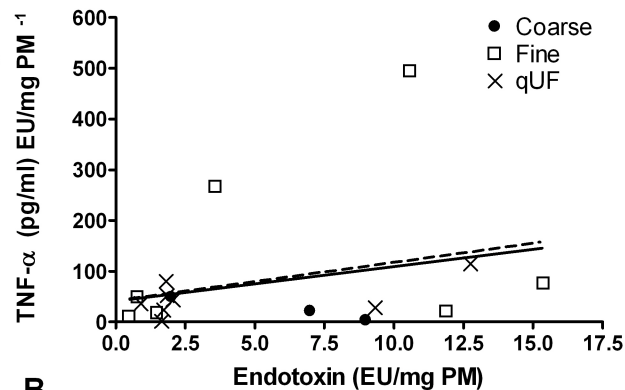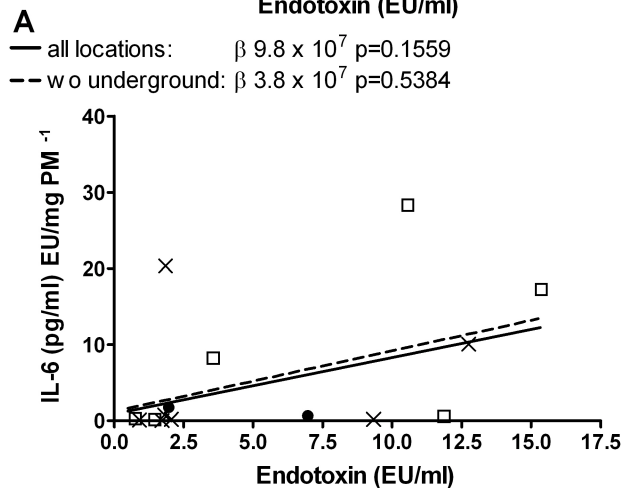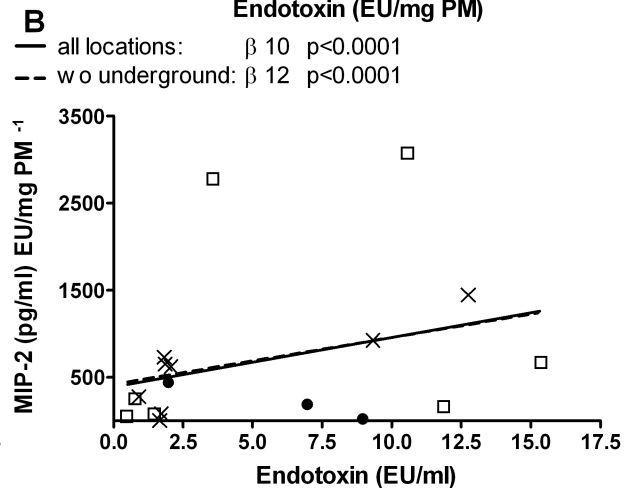

Supplement: Additional file 6 — Figure s1. Relationship between the particulate matter (PM) endotoxin content and cellular responses in RAW 264.7 macrophages. Cells were exposed to increasing concentrations of PM after which MTT-reduction activity and the release of pro-inflammatory markers was measured. For each cellular parameter, the slope of the concentration-response curve is plotted against the corresponding endotoxin content of each PM sample. The solid lines represent correlation investigated for all sites, dotted lines without the underground railway station site (wo underground). Panel A: MTT- activity (n = 24 PM samples; 8 sites × 3 PM size fractions). Panel B-D: release of pro-inflammatory markers (n = 18 PM samples, since 6 were excluded because of high endotoxin levels). Statistical analysis was performed by multiple linear regression and shown as effect estimate (β, slope) and belonging p-value. [file 1743-8977-8-26-S6.PDF]

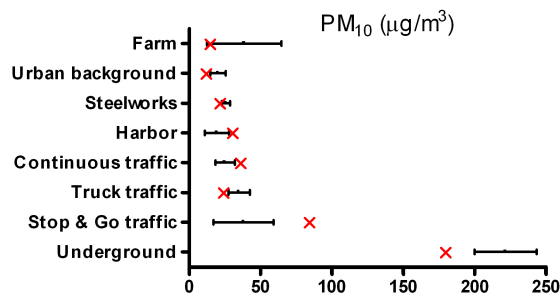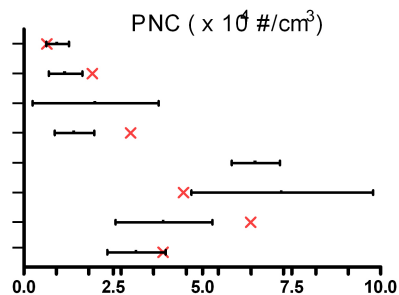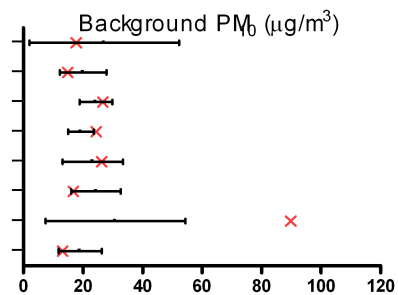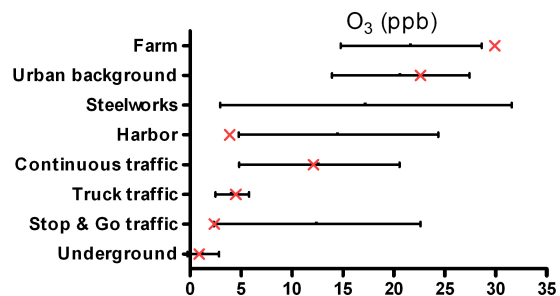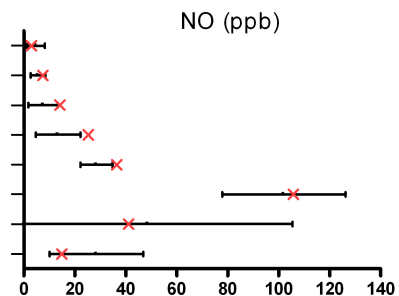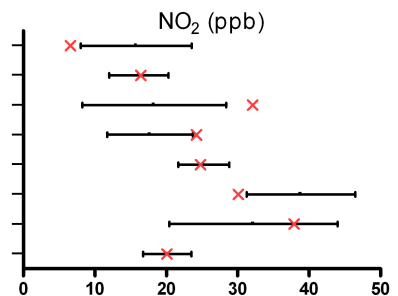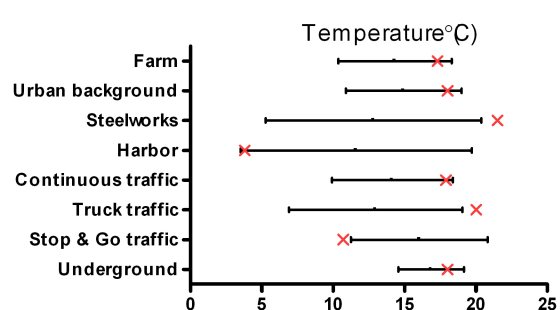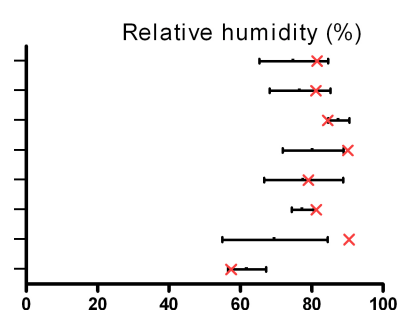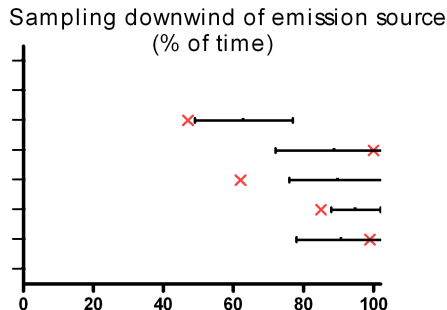

Supplement: Additional file 8 — Figure s2. Mean values of air pollutants and meteorological conditions of the selected sampling day (red crosses) compared to the site average (black squares with standard deviation bars). PM sampling was carried out at eight sites for 4 - 12 separate days and for 6 hours per day at each site. Per site, the sampling day with highest PM concentration was chosen for in vitro toxicity testing to ensure sufficient availability of material for the various tests (selected days had highest coarse, fine and quasi UF particle concentrations, data not shown). Sampling at the farm, urban background and underground sites was independent from wind direction. PNC values were not available for the selected days of the steelworks and continuous traffic site. Background levels of PM10 were obtained from the Dutch National Air Quality Monitoring Network. PNC, particle number concentration (median). [file 1743-8977-8-26-S8.PDF]
