# Supplementary material for: In vitro toxicity of particulate matter (PM) collected at different sites in the Netherlands is associated with PM composition, size fraction and oxidative potential - the RAPTES project
Source: Part Fibre Toxicol. 2011 Sep 2;8:26. doi: 10.1186/1743-8977-8-26 (PMC3180259; doi:10.1186/1743-8977-8-26)
Supplement: Additional file 7 — Table s5. Relationship between the PM oxidative potential (DTT consumption) and cellular responses in RAW 264.7 macrophages exposed to PM collected at eight contrasting sites. Cells were exposed to increasing concentrations of particulate matter (PM) after which MTT-reduction activity and the release of pro-inflammatory markers was measured. For each cellular parameter separately, the slope of the concentration-response curve was plotted against the corresponding DTT consumption of each PM sample. Subsequently, multiple linear regression was used to calculate the associations (β, slope, and belonging p-value) between DTT consumption and cellular responses. Data on DTT consumption was missing for four samples (1 coarse and 3 qUF). Furthermore, six PM samples were excluded from data analysis with regard to the pro-inflammatory responses because of high endotoxin levels (5 coarse and 1 fine sample; one of the coarse samples was also lacking information on DTT consumption). The coarse fraction was not included in the data analysis on the pro-inflammatory markers (TNF-α, IL-6 and MIP-2), since n ≤ 3. n = number of sites included. wo underground, without underground railway station site. Bold values indicate statistically significant associations (p < 0.05). [file 1743-8977-8-26-S7.PDF]

	**MTT-reduction activity (%) μg^{-1} PM (p-value) n**	**TNF-α (pg/ml) μg^{-1} PM (p-value) n**	**IL-6 (pg/ml) μg^{-1} PM (p-value) n**	**MIP-2 (pg/ml) μg^{-1} PM (p-value) n**
All size fractions				
all sites	**-0.850 (<.0001) n=20**	45.3 (0.2430) n=15	-1.43 (0.7256) n=15	674 (0.0884) n=15
all sites wo underground	**-1.45 (<.0001) n=17**	**1.53×10^3 (<.0001) n=12**	**79.7 (<.0001) n=12**	**111×10^3 (<.0001) n=12**
By size fraction				
coarse	**-0.963 (<.0001) n=7**	n=3	n=3	n=3
coarse wo underground	-3.204 (0.0867) n=6	n=2	n=2	n=2
fine	**-0.916 (<.0001) n=8**	28.9 (0.7384) n=7	-8.33 (0.2607) n=7	31.7 (0.9661) n=7
fine wo underground	**-2.05 (<.0001) n=7**	**2.27×10^3 (<.0001) n=6**	**87.3 (0.0004) n=6**	**169×10^3 (<.0001) n=6**
qUF	**-0.778 (<.0001) n=5**	-7.92 (0.7195) n=5	-2.33 (0.4581) n=5	2.74×10^3 (<.0001) n=5
qUF wo underground	-0.927 (0.0844) n=4	**216 (0.0122) n=4**	1.66 (0.8798) n=4	**3.80×10^3 (<.0001) n=4**
